# How the size of the to-be-learned material influences the encoding and later retrieval of associative memories: A pupillometric assessment

**DOI:** 10.1371/journal.pone.0226684

**Published:** 2019-12-31

**Authors:** Péter Pajkossy, Mihály Racsmány

**Affiliations:** 1 Institute of Cognitive Neuroscience and Psychology, Research Centre for Natural Sciences, Budapest, Hungary; 2 Department of Cognitive Science, Budapest University of Technology and Economic, Budapest, Hungary; University of Birmingham, UNITED KINGDOM

## Abstract

Pupillometry have recently added valuable insights about the cognitive and possible neurobiological processes underlying episodic memory. Most of the studies, however, investigated recognition memory, which only partially relies on cue-driven recollection, the hallmark feature of episodic memory. Here we measured pupil size during a paired associate learning task, where participants encoded word-pairs, and after a short delay they took part in a cued recall. Importantly, we manipulated the size of the learning set: participants either learnt two, four or eight word-pairs in a row. As expected, increasing set size resulted in larger forgetting, assumingly as a consequence of weaker memory strength for the word-pairs. Our results show an important difference between pupil size changes observed during encoding and retrieval. During retrieval, the pupil instantly begun to dilate, as a sign of increased processing load accompanying the retrieval of the target memory. Importantly, large set size was also associated with larger pupil dilation during retrieval. This supports the notion that pupil dilation can be regarded as a marker of memory strength. In contrast, during encoding, pupil dilation decreased with increasing amount of encoded information, which might be due to the overuse of attentional resources. Furthermore, we also found that serial position during encoding modulated subsequent memory effects: for the first three serial positions, successful recall was predicted by larger pupil dilation during encoding, whereas such subsequent memory effect was absent for later serial positions. These results suggest that the amount of information independently modulates pupil dilation during encoding and retrieval, and support the assumption that pupillometric investigation of paired associate learning could be an informative way to investigate the cognitive and neurobiological processes of episodic memory.

## Introduction

Since the pioneering work of Hess [1] and Kahneman and Beatty [2], there is a growing amount of evidence suggesting that the size of the pupil tends to change as a consequence of information processing [3,4]. These changes have been successfully linked to various psychological concepts, like mental effort [5], surprise [6], controlled attention [7], working memory [8] or the trade-off between exploiting current rewards vs. exploring new sources of reward [9,10]. The diversity of the results might be explained by the fact that pupil size is correlated with the activity of the locus coeruleus (LC) [11,12], a small brain stem nucleus, from which the majority of cortical noradrenergic (NA) projections originate. This so called LC/NA system is suggested to coordinate task-relevant cortical networks, and has an extensive influence on cortical information processing [13,14].

### Pupillometric investigation of recognition memory

Due to this general link between pupil size and arousal systems of the brain, pupillometry might be a useful tool to investigate episodic memory, our ability to recollect and reconstruct past experiences [15,16]. During encoding, different parts of the experiences are bound together and stored as an episodic memory trace, and this trace can be recalled when an appropriate retrieval cue activates that past memory [15–18].

The application of pupillometry in relation to memory consists of overwhelmingly recognition memory studies. In recognition tasks, participants first see a set of items during the encoding phase (old items), which are then presented again intermixed with items previously not seen (new items). Participants then have to decide whether the presented item is old or new. In such tasks, it has been repeatedly found that old responses, as compared to new responses, are either associated with higher pupil dilation [19–23], or by attenuated constriction of the pupil [24]. Based on such results, several authors [19,20,24] concluded that the pupil old-new response might be compared to the different event-related potentials (ERPs) observed for old versus new words, which is suggested to index memory strength or prior occurrence [25]. This assumption was supported by findings showing that the confidence level of correct recognition judgements positively correlated with pupil dilation [20] and also by results demonstrating higher pupil dilation for correct old judgements after deep than shallow encoding of words [21]. In contrast, Bradley and Lang [22] found that item repetition independently modified pupil dilation and old-new ERPs. Based on this result, the authors suggested that pupil dilation is not associated with memory strength. Instead, it can be viewed as an aggregate measure of other task-related factors, such as difficulty or subjective confidence.

The investigation of pupil size changes during encoding of memories led also to inconsistent findings. An established method for studying psychophysiological correlates of encoding is to compare the encoding phase of later successfully retrieved items with later forgotten items (see e.g. [26]). These so called *subsequent memory* effects are inconsistently related to pupillometric data in recognition memory studies. For instance, Papesh, Goldinger and Hout [20] reported higher pupil dilation during encoding of later recalled as compared to later forgotten items, whereas no difference was found in other studies [19,22]. In contrast, Kafkas and Montaldi [27] found that lower pupil dilation was associated with later successfully recognized items, and Naber, Frässle, Rutishauser and Einhäuser [24] reported that during encoding of later remembered pictures there was a larger pupil constriction in comparison to later forgotten pictures.

Despite these broad set of data, methodological differences make it difficult to interpret the findings. For example, some of the studies used incidental, whereas other studies used intentional encoding, which might have a large effect on the recruited cognitive processes. As Papesh et al [20] note, differences between subsequent memory effects might be related to different encoding instructions.

Importantly, the use of recognition memory tasks provides some shortcomings in unfolding the relationship between pupillometric changes and episodic memory processes. Recognition memory judgements are suggested to be influenced by two processes: a general sense of familiarity and the recollection of specific details of previous encounters ([28], although see [29] for unequal-variance signal-detection explanations of recognition). These processes are sensitive to different factors during both encoding and retrieval, and they influence the size of the pupil old-new effect (i.e. recollective old responses elicit higher pupil dilation than familiarity responses, e.g. [21]). Nevertheless, their respective contribution to the pupil-old new effect is not always assessed: in several studies, only an old-new decision is required from the participants, and no method is used which could disentangle recollection and familiarity based decisions ([19,20,22], but see [21]). Furthermore, due to the dual-process nature of recognition memory, it only partly relies on episodic recollection and item-context (cue) binding, the hallmark features of episodic memory [15,16].

### Pupil size changes during cued and free recall tasks

To investigate recollection and binding in the context of episodic memory, cued or free recall tasks are better suited: in these procedures, unlike a recognition memory task, the target item itself is not presented during the test. Instead, the reconstruction of the original memory is required either with the help of some externally presented cue (cued recall) or without any help (free recall). When compared to findings of recognition memory, pupillometric investigations of free and cued recall show both similarities and differences.

For example, in a recent study, Bergt, Urai, Donner and Schwabe [30] showed that subsequent memory effects can be also demonstrated, when the final test is free recall and the delay period is long: participants first were asked to memorize a series of items and were then asked to recall the items immediately and also after a 24-hour delay. The results show that recall success on both tests was associated with higher pupil dilation during the encoding phase.

Similar result was reported by Kucewicz and colleagues [31]: larger pupil dilation during the encoding of words was associated with recall success in a free recall task following a short delay. In this study, the authors also examined pupil size changes during the test phase and found that increased pupil size characterizes the time window before and after the recall of a word. Memory strength, however, was not manipulated in this study, thus it remains to be tested, whether pupil size during free recall is sensitive to this factor.

Finally, Van Rijn, Dalenberg, Borst and Sprenger [32] investigated pupil responses during a paired associate learning task. They varied the memory strength of item-item associations by repeatedly retrieving a set of paired associates (names and location of brain areas). Furthermore, they also varied the size of the set to be repeated, thereby manipulating the retention interval between two retrievals. Both factors, repetition and retention was suggested to increase memory strength, and the authors found that both were associated with a reduced pupil dilation during retrieval. This is somewhat contradictory to the results of recognition studies, where memory strength was related to higher pupil dilation during recognition [20,21]. This contradiction, in line with a plethora of other behavioral results [33,34], shows that recognition memory and cued recall tasks index different aspects of episodic retrieval, and thus the relationship between memory strength and pupil size changes might differ depending on which task is used. Because of this, research on pupil dilation during free and cued recall tasks is warranted, and the present research aims to contribute to this growing research area by examining pupil size changes during a cued recall task.

### The present study: Pupillometric investigation of paired associates learning

One widely used cued recall task is paired-associate learning, where participants have to learn the association between two independent items (e.g. two words), and they are later presented with one of the items, the cue (e.g. the first word), and have to recall the target item (e.g. the second word). Thus, during encoding, participants might create episodic traces by binding the items, and during cued recall, these traces have to be assessed.

We constructed a paired-associate learning task, which consisted of alternating runs of encoding and recall blocks. Memory strength and processing load were manipulated by the size of the learning set: participants either learnt 2,4, or 8 word-pairs. Larger set size was considered to be associated with weaker memory strength: during both encoding and retrieval of paired associates, larger set size was considered to trigger larger interference, which might hinder the encoding of the paired associate and also induce interference during retrieval. In line with Van Rijn et al [30] work, we hypothesized that smaller set size will be associated with higher memory strength. Consequently, the retrieval of these items will require less processing resources, and therefore it will be associated with smaller pupil dilation.

Furthermore, subsequent memory effects during encoding were also investigated. Due to the alternating nature of encoding and recall blocks, participants were aware that all the presented items have to be retrieved later, thus the encoding was intentional. In previous research, during intentional encoding of words, higher pupil dilation was associated with correct recognition [20] and with recall success [30,31]. Based on this, we predicted that higher pupil dilation will be associated with the encoding of later correctly recalled items, as compared to later forgotten items. This hypothesis was also based on the fact that intentional encoding is characterized by effortful encoding operations triggering activity of the LC/NA system (processing of the words, search the semantic memory for potential associations, etc), and so might dilate the pupil.

For analyzing pupil dilation responses associated with subsequent memory in our paradigm, we also took in account that pupil dilation during the encoding phase can be affected by serial position. In a recent study, Miller, Gross, Unsworth [35] found that serial position during encoding of single words influenced task-evoked responses. Because of this, we also examined whether pupil dilation linked to subsequent memory is affected by serial position during encoding.

## Method

### Participants

Data were collected from 38 participants, 17 females, *M*_age_ = 22.79 years, *SD* = 2.13, range = 19–28. Participants were undergraduate students paid for their participation. The research project was approved by the United Ethical Review Committee for Research in Psychology, Hungary (approval number: 2019–11). Participants gave written informed consent.

### Material

Using a Hungarian word frequency norm [36], we selected 96 low-frequency words and created 48 word-pairs (see S1 Appendix). The words contained two syllables and were five or six letters long. Word-pairs were matched randomly, but we avoided word-pairs with clear semantic or phonemic relations between the pairs. Semantic relatedness was tested in a pilot study (*N* = 42): we asked participants to rate several word-pairs on a nine-point scale of semantic relatedness (1: semantically distinct 9: semantically similar). The mean score of semantic relatedness for the final set of word-pairs was *M* = 1.42 (*SD* = 0.34).

The memory task was conducted using the software Presentation (Neurobehavioral Systems Inc, Albany, CA). Pupil size was recorded using an SMI HiSpeed 1250 tower-mounted eyetracking system (SensoMotoric Instruments, Teltow, Germany). Data sampling frequency was 1250 Hz. Pupil data were processed using MATLAB (MathWorks, Natick, MA, USA).

### Design

A simple memory task was constructed, in which participants took part in several blocks of paired-associate learning with subsequent cued recall. In each block, participants first learned several word-pairs (e.g. cotton—kayak). Subsequently, after a delay of 20 seconds, they were presented with the left-side word of each word-pair, the cue word (e.g. cotton—?), and were required to retrieve its pair, the target word (e.g. kayak). The presentation order was fully randomized independently for the word-pairs during encoding and the cue-words during retrieval.

As experimental manipulation, we varied the size of the learning set by manipulating the number of word-pairs in each block. We used three different block-types with different size and frequency: eight blocks with two word-pairs (small set size condition), four blocks with four word-pairs (medium set size condition), and two blocks with eight word-pairs (large set size condition). Thus, for each experimental condition with different set size, 16 words-pairs were tested. The assignment of word-pairs to experimental condition, the sequence of the word-pairs in a given block, and the sequence of the blocks was randomized.

### Procedure

Participants were tested individually in a dimly lit room. First, we asked them to place their head in the eye-tracker and adjusted a head microphone so that the exact time of the oral response could be recorded. The experiment started with written and oral task instructions, and was followed by a calibration procedure for the eye-tracker.

The background color of the screen was light grey, and the words were presented with black font color. During the whole experiment, lines with dark grey colors designated a rectangle at the center of the screen (size: 6.6° x 27.4° in visual angles). Because measured pupil size might vary as a function of gaze position, we asked participants to restrict their gaze movement to this area during the experiment.

Each block had an encoding and a recall phase. The start of the encoding phase was indicated by the label ‘Study’, presented for 3 seconds on the screen. Thereafter, the word-pairs (two, four or eight, depending on the set size condition) were shown subsequently, each word-pair for five seconds. To enable the pupils to return to baseline levels, after each word-pair, a mask stimulus was presented for another five second. This mask stimulus was a group of nonsense characters, presented at the center of the screen (‘!*%%/*!*%—“+%/*!*%%/%*’). With this display setup, the visual features of the mask stimulus were made similar to the subsequent word presentation display, and so the size of the pupil was less affected by low-level visual attributes. After the last to be remembered word-pair in the block, a delay phase of 20 seconds followed. During the delay, participants saw 3-digit numbers, and had to indicate with a keypress whether the sum of the digits was divisible by three or not. After the delay, the recall phase was indicated by presenting the caption ‘Test’ on the screen for 3 seconds. Thereafter, each cue-word was shown for five seconds, followed by a mask stimulus presented also for five seconds. The same mask stimulus used in the learning phase was used to ensure that the pupil dilation is not influenced by effects related to the previous trial. The cue-word was always shown for five seconds, regardless of whether or not the participant produced a response.

During recall, participants were instructed to say out aloud the to-be-retrieved target word without removing their gaze from the screen. Using a head-microphone, we measured response time and the experimenter checked on a sheet containing all word-pairs whether the retrieved word was correct or not and registered the response. To facilitate this task, we assigned a number to each word-pair (fixed for all participants), and this number was presented together with the cue-word during the test trial (on the top of the screen). We told participants, however, not to attend to this number, as it is only to facilitate the experimenter’s work (every number was only shown once, and participants were not aware of the matching between word-pairs and numbers, thus they could not use this information in any task-relevant way). After half of the blocks, a short break was inserted, which was followed by a recalibration of the eye-tracker.

Importantly, we did not inform participants before the encoding phase how many items the given learning set will contain. With this manipulation, we aimed to enforce participants to always focus on the encoding of the current word-pair, and decrease the influence of different metacognitive strategies.

### Data processing

Mean recall percentage and mean reaction time for each set size condition was computed. Reaction time was recorded by a built-in voice recording function of the experiment builder software: it was recorded when the sound intensity exceeded a predefined threshold. With this method, we were able to capture the start of the oral response in most cases. Due to false alarms caused by miscellaneous noises (e.g. sighs or coughs), the reaction time could not be determined for a minority of the trials. Out of the 48 test trial, reaction time data was lost on average for 6.64 trials (SD = 7.63, range: 0–34). There were five participants with excessive data loss (no reliable reaction time data for more than 15 test trials). Because such data loss was not expected to influence our results in a systematic way, we did not exclude those participants from RT data analysis. Nevertheless, we run our analyses also without these five participants, and the results were similar (see S1 Text).

Pupil size was measured in pixels (i.e. the number of pixels occluded by the pupil in the video image of the eye-tracker). Always the pupil size of the right eye was measured. The pre-processing of pupil data was performed using a computer algorithm written in MATLAB. Because changes in pupil size are relatively slow, the original data were down-sampled to 125 Hz before further processing. We used the horizontal diameter of the pupil in our computations, because some of the participants tended to drop their eye-lid during the task which then covered the upper part of the pupil, introducing noise into the measurement of the vertical diameter.

To identify sources leading to noise in the pupil signal, we first used the built-in blink detection algorithm of SMI and removed all data points, which were identified as blinks (ratio of removed data points: *M* = 7.03%, *SD* = 4.95). Then, to filter out noise caused by the movements of the eyelid before and after blinks, we excluded the 60 msec before and after each blink (ratio of removed data points: *M* = 4.21%, *SD* = 2.78). Thereafter, further data points with the value of zero were removed (ratio of removed data points: *M* = 0.15%, *SD* = 0.40).

After the removal due to blinks and other artefacts, we separated the data into 96 short data sequences, each data sequence containing either a mask trial and a subsequent encoding trial (48 sequences) or a mask trial and a subsequent recall trial (48 sequences). We calculated the mean and standard deviation for each of these data sequences, and removed all data points, which were more than 2.5 standard deviations above the mean pupil size in a given data sequence (ratio of removed data points: *M* = 0.23%, *SD* = 0.10).

Overall, 11.62% (SD = 7.52) percent of all data points were removed. The missing data points were interpolated. After interpolation, the data were smoothed using the Savitzky-Golay filter (parameters: polynomial order:2, frame size:21).

Additionally, we also scanned all 96 data sequences of each participant to identify individual trials with excessive amount of removed data, and dismissed trials with over 30% of interpolated data points. For three participants, an excessive amount of data sequences had to be removed (at least 34 data sequence out of 96). Due to poor data quality, we removed data for these three participants from our analyses. For the remaining sample (N = 35, 16 female, *M*_age_ = 22.75 years, *SD* = 2.12, range = 19–28), on average 2.86 items had to be excluded (SD = 4.51, range 0–17, 3% of all data sequences). The amount of data samples affected by the different steps of data filtering can be found for each participant in S1 Dataset.

After data filtering, each of the 96 sequence was standardized using the mean and standard deviation of that sequence. Finally, to control for within-task variation of pupil size, all trials were baseline corrected: First, we calculated the mean pupil size during the 500 msec preceding each encoding or recall trial (i.e. the last 500 msec of the preceding mask trial). Then, we subtracted this mean value from each data point of the trial. With this method, we could calculate the change compared to the pretrial baseline.

### Statistical analysis

First, we used the Kolmogorov-Smirnoff test to investigate whether our dependent variables are normally distributed. In the case of non-normal distribution, nonparametric statistical methods were used.

The effect of set size on reaction times was investigated using a repeated measures ANOVA, whereas for testing the effect of set size on recall percentage, the nonparametric Friedmann-test was used.

To analyze changes in pupil size, we differentiated between an early and a late response for each encoding/test period (for a similar approach, see [22]). Research using ERP analysis showed that the first ERP components associated with recollection are emerging no sooner than 500 msec (e.g. [37], for a review see [38]). Furthermore, there is a time-lag of about 200–400 msec between LC firing and the accompanying pupil response [12]. Consequently, pupil size changes in the first second after stimulus presentation might reflect LC/NA responses associated with perceptual and attentional orienting processes (see e.g. [39,40]) To capture these changes, we computed the mean value of pupil size during this early response period (0–1000 msec after stimulus onset) for each trial of each participant. This first measure was termed *early pupil response*. During the late response period (1000–5000 msec after stimulus onset), neural activity associated with encoding and retrieval is supposed to be the dominant source affecting pupil signal. To quantify this late pupil response, we calculated the mean pupil size during this late response period (1000–5000 msec after stimulus onset) for each trial of each participant. This second measure was termed *late pupil response*. In each of the analysis described below, one of these two variables was used as dependent variable.

First, we investigated early and late pupil responses during both encoding and recall, to see how these mental processes affect pupil size. During retrieval, only trials with correct responses were involved in the analysis. Then, we conducted additional, more detailed analyses focusing on how different aspects of encoding and retrieval affect the observed pupil response.

For the recall blocks, we investigated whether early and late pupil responses differed for the different set size conditions. To this analysis, we used a one-way repeated measures ANOVA with set size, as within-subject factors. Here again, only correct retrieval trials were analyzed.

We also conducted an additional analysis to exclude the confounding role of output interference for the retrieval trials (i.e. interference resulting from previously recalled items in the same block). The three conditions differed in the amount of information retrieved during a block, thus the amount of output interference should differ in the three conditions with interference being the most prevalent in the large set size condition. This might confound pupil size changes, because control processes activated to overcome output interference might also dilate the pupil. Thus, higher pupil dilation might not be related to set size per se, but might be a consequence of the higher interference generated during longer test phase. To exclude this alternative explanation, we analyzed our data also by excluding the last four test trials from the large set size condition, to equal the amount of output interference between the medium and the large set size condition. One participant had no successful recalls from the first four retrieval trials of the large set size condition, thus his/her data were not involved in this particular analysis.

We used paired sample t-tests to investigate subsequent memory effects. We compared both early and late pupil size changes during encoding of later successfully retrieved and later forgotten items, respectively. To control for any confounding effects of serial position, we computed early and late pupil responses separately for each serial position and investigated the effect of serial position on pupil dilation by using a repeated measures ANOVA with serial position, as within subject factors (1st to 8th serial position).

In all ANOVAs, when the assumption of sphericity was violated, the Greenhouse-Geisser adjusted *F*-value was computed. To compare the experimental conditions, we used contrast analysis to directly compare the subsequent levels of our independent variables. Raw data are presented in S2 Dataset.

## Results

### Behavioral data

**Fig 1:**
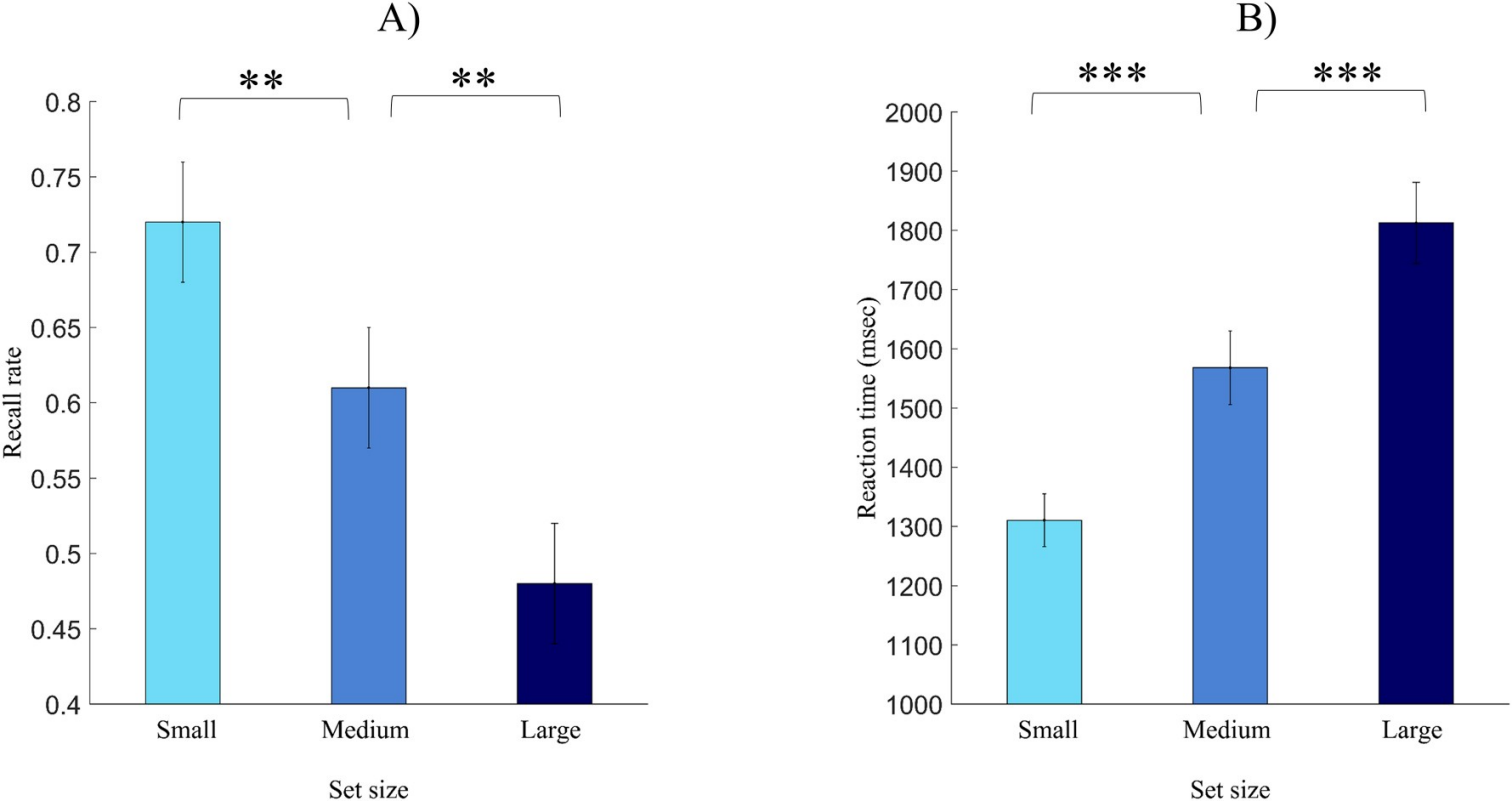
Behavioral data in the recall phase. Recall rate (A) and reaction time of recall (B) for the small, medium and large set size condition, respectively. Error bars represent standard error of the mean. **: p < .01; ***: p < .001.

Recall percentage and reaction time data for the three conditions are presented on Fig 1A and 1B. As recall percentage for the small and large set size condition was not normally distributed, we used the nonparametric Friedman-test to show that there is a significant difference in recall performance for the three groups, χ^2^ (2) = 22.23, *p* < .001. Post-hoc tests using the Wilcoxon signed rank test showed that there is a significant difference between the small and the medium, and also between the medium and the large set size condition (*Z* = 2.89, *p* = .004, *r* = .28; and *Z* = 3.20, *p* = .001, *r* = .31 respectively).

A repeated measures ANOVA with set size as within-subject factor showed a significant main effect for reaction time, *F* (1.2,41.1) = 27.38, *p* < .001, η_p_^2^ = .45, as dependent variable. Planned contrast analyses showed that the three set size conditions differed from each other significantly (small vs. medium: *F* [1,34] = 111.73, *p* < .001, η_p_^2^ = .77; medium vs. large: *F* [1,34] = 27.32, *p* < .001, η_p_^2^ = .45). These data show that our memory strength manipulation was successful.

### Pupil size changes during encoding and recall

**Fig 2:**
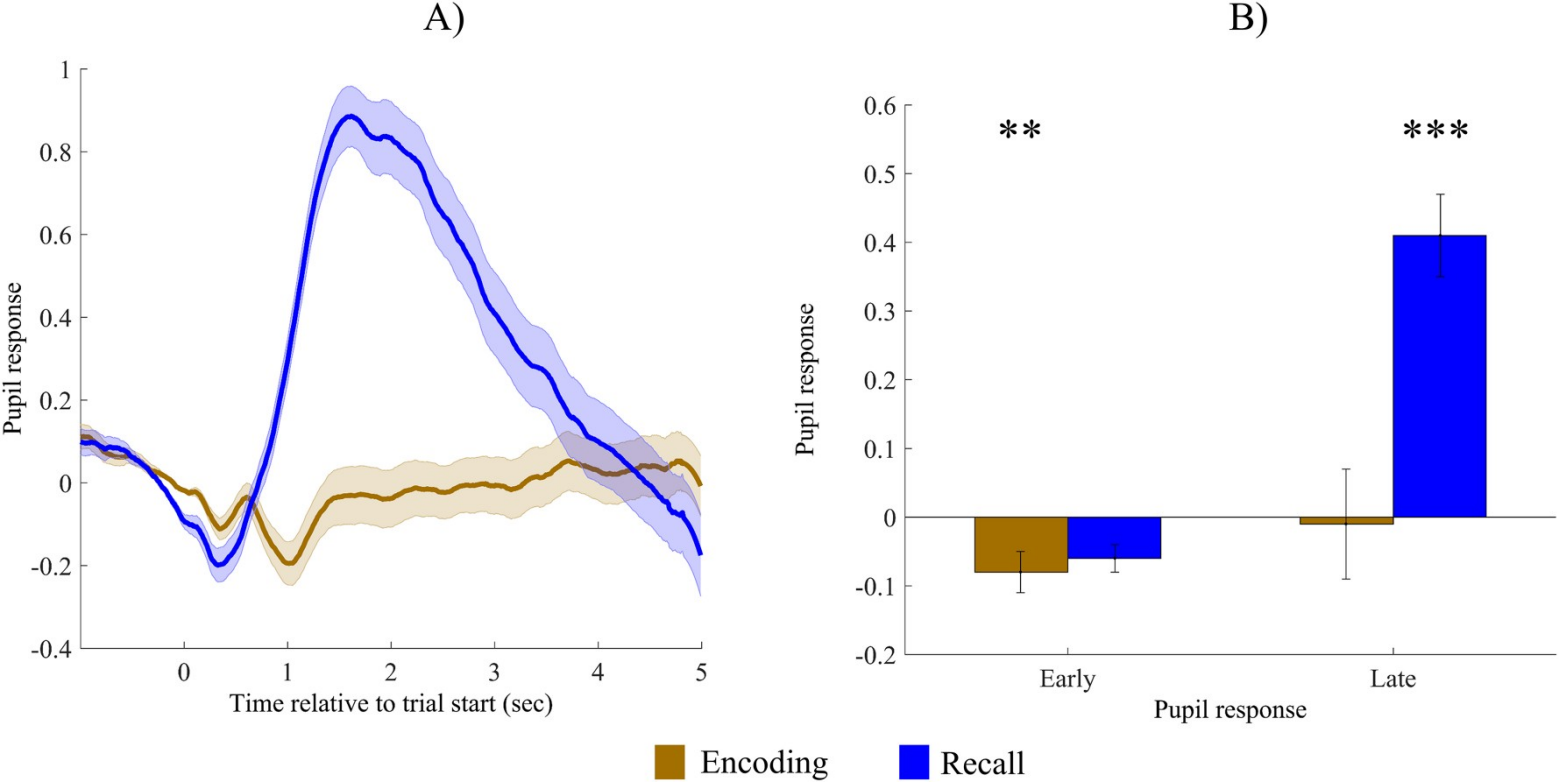
Pupil size changes during encoding and recall of paired associates. (A) Pupil size changes, depicted separately for the encoding and correct recall trials. Time is shown relative to the start of the trial. Standardized and baseline corrected values are presented: the mean of the 500 msec preceding trial onset is subtracted from each data point. (B) Early and late pupil response, presented separately for the encoding and correct recall trials. Annotations of significance refer to the results of one sample t-tests with a test value of 0. Error bars and shaded error regions represent standard error of the mean. **: p < .01; ***: p < .001.

Fig 2A depicts pupil size changes during the encoding and successful recall of paired associates. As can be seen, retrieval is associated with a large dilation of the pupil starting 500 msec after the presentation of the cue, whereas during encoding trial, a constriction of a smaller magnitude begins with similar timing, and the pupil then returns to its baseline level. We used a one-sample *t*-test with a test value of 0 to test whether early and late pupil responses are significantly different from baseline (early and late pupil responses are depicted on Fig 2B). During encoding, the pupil significantly constricted during the first 1000 msec (*M* = -0.08, *SE* = 0.02, *t*(34) = 3.38, *p* = .002, *d* = 0.54), whereas it returned to its baseline levels thereafter, so late pupil response was not different from baseline after the first second (*M* = -0.01, *SE* = 0.06, *t*(34) = 0.17, *p* = .87, *d* = 0.02). During successful recall of paired associates, there was a nominal decrease in pupil size during the first 1000 msec, but this change was not significant (*M* = -0.06, *SE* = 0.03, *t*(34) = 1.70, *p* = .09, *d* = 0.29). In contrast, compared to baseline, pupil size increased significantly after the first second (M = 0.41, SD = 0.07, *t*(34) = 5.31, *p* < .001, *d* = 0.89).

To sum up, the beginning of the encoding phase was accompanied by a small but significant constriction, which was observable during the first 1000 msec. For the retrieval phase, the pupil showed a marked dilation during retrieval, which emerged after 500 msec.

### Set-size dependent pupil size changes during recall

**Fig 3:**
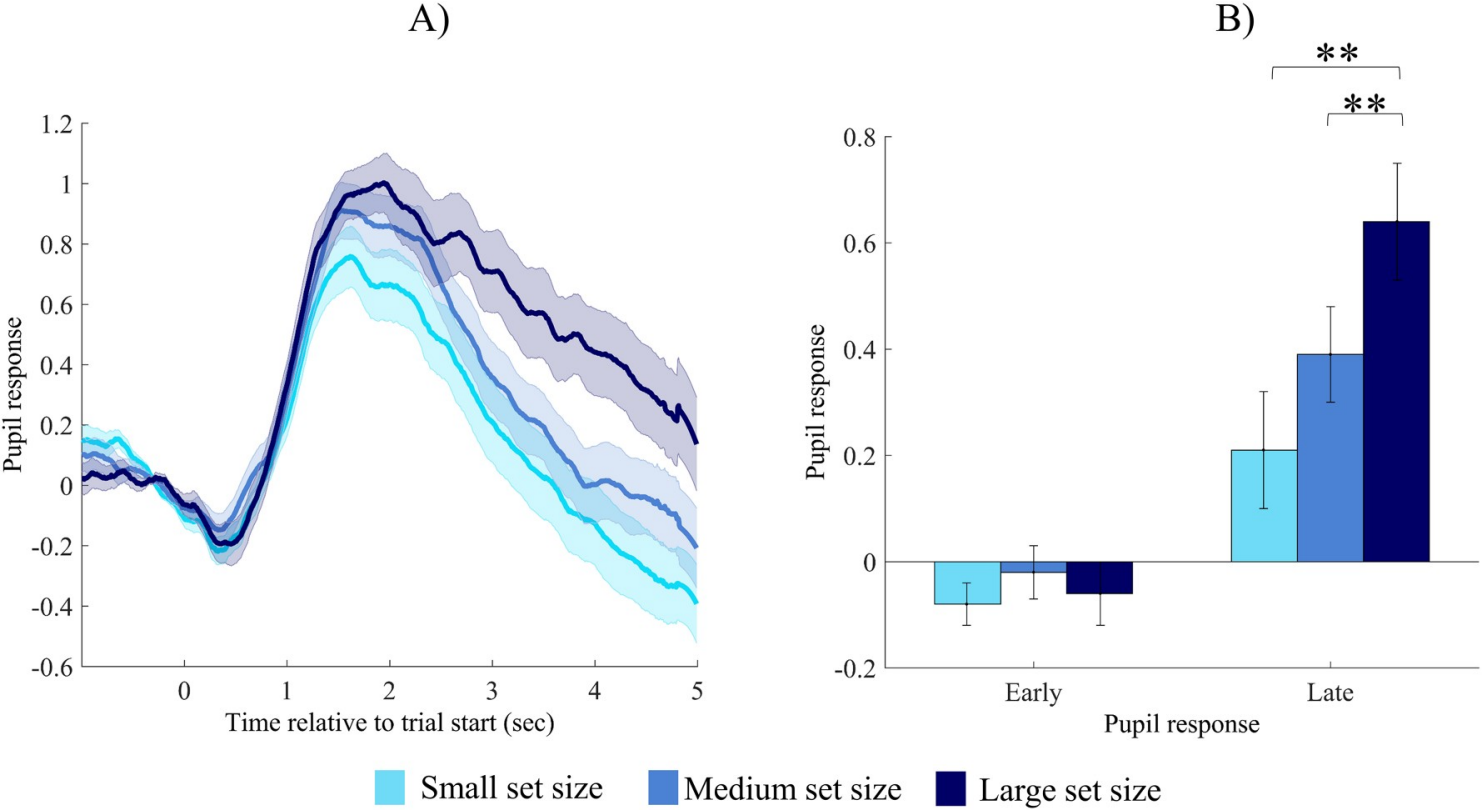
Pupil size changes during recall of paired associates. (A) Pupil size changes during recall of items, depicted separately for the small, medium and large set size condition, respectively. Values are standardized and baseline corrected: the mean of the 500 msec preceding trial onset is subtracted from each data point. Time is shown relative to the start of the trial. (B) Early and late pupil responses or the small, medium and large set size condition, respectively. Error bars and shaded error regions represent standard error of the mean. ** p < .01.

Pupil size during recall trials in the three conditions for correctly retrieved words is shown in Fig 3A. As can be seen, the three set size conditions elicit different pupil responses. This observation is validated by a one way repeated measures ANOVA with set size (small-medium-large) as within subject factor, and early and late pupil response, respectively, as dependent variables (see also Fig 3B). For early pupil response, we found no significant difference between different set sizes, *F* (2,68) = 0.73, *p* = .48, η_p_^2^ = .02. In contrast, late pupil response differed for the three set size conditions, *F* (2,68) = 7.69, *p* < .001, η_p_^2^ = .18. Planned comparisons using repeated contrasts showed that the pupil dilated more during retrieval in the large set size condition, than in the medium set size condition, *F* (1,34) = 8.50, *p* = .006, η_p_^2^ = .20. Retrieval from the medium set size condition elicited nominally higher pupil dilation, than retrieval from the small set size, but this difference was not significant, *F* (1,34) = 2.40, *p* = .13, η_p_^2^ = .07. Finally, in a post-hoc analysis we also contrasted late pupil response of the small and the large set size condition, respectively. A simple contrast with the small set size condition, as reference category revealed a significant effect, *F* (1,34) = 11.86, *p* = .002, η_p_^2^ = .26. This means that successful retrieval in the large set size condition evokes larger pupil response than retrieval in both the medium and the small set size conditions.

Finally, to exclude the alternative hypothesis of the effect of interference on encoding, we ran the previous analysis by only involving the first four test trials of the large set size condition in our analysis. The magnitude of the late pupil response remained similar (*M* = 0.66, *SE* = 0.13), and the pattern of the results did not change either: there was a significant main effect of the set size factor, *F* (1.5, 49.2) = 8.43, *p* = .002, η_p_^2^ = .20. The contrast analysis showed that there is a significant difference between the medium and the large set size condition, *F* (1,33) = 11.24, *p* = .002, η_p_^2^ = .25, and also between the small and the large set size condition, *F* (1,33) = 10.76, *p* = .002, η_p_^2^ = .25.

To sum up, retrieval of items from the large learning set evokes higher pupil dilation, as compared to the medium or the small learning set, and this difference cannot be attributed to output interference.

### Subsequent memory analysis

**Fig 4:**
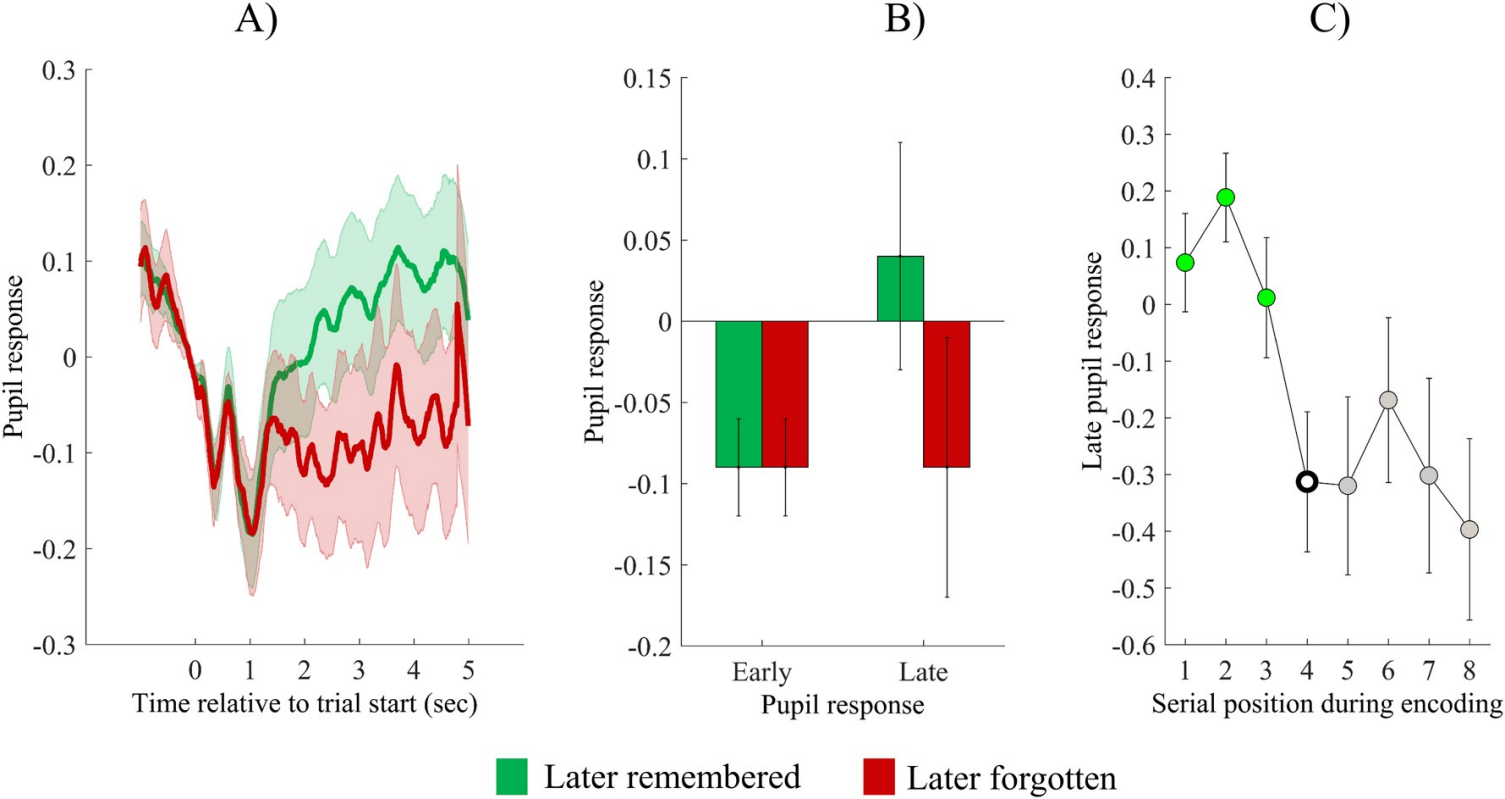
Pupil size changes during encoding: Serial positon and subsequent memory effects. (A) Pupil size changes during encoding of items, depicted separately for later recalled (green line) and later forgotten items (red line), respectively. Values are standardized and baseline corrected: the mean of the 500 msec preceding trial onset is subtracted from each data point. Time is shown relative to the start of the trial. (B) Early and late pupil responses for later recalled and later forgotten items, respectively. (C) Late pupil response as a function of serial position. Green markers denote significant difference from the fourth serial position (hollow marker). Grey markers indicate no significant difference. Error bars and shaded error regions represent standard error of the mean.

To investigate pupil size changes during the encoding of later recalled versus later forgotten items, we calculated early and late pupil response measures for both conditions, and compared them using a paired t-test. As can be seen on Fig 4A and 4B, we found no difference for the early pupil response, *t*(34) = 0.24, *p* = .81, *d* = 0.02, and there was a nonsignificant tendency for larger pupil dilation during encoding of later successfully recalled items, *t*(34) = 1.78, *p* = .08, *d* = .29.

Thereafter, to control for possible confounding effects, we investigated how early and late pupil responses during encoding change as a function of encoding position. We used a repeated measures ANOVA with serial position as within subject factor (1^st^ to 8^th^) and either early or late pupil response, as dependent variable. We found a significant main effect of serial position on the late pupil response, *F* (4.78,162.57) = 3.13, *p* = .011, η_p_^2^ = .08, whereas no significant effect was found for the early response, *F* (4.78,162.776) = 1.78, *p* = .12, η_p_^2^ = .05. As can be seen on Fig 4C, late pupil response decreased with increasing serial position, starting from the fourth serial position. To support this impression, we used simple contrasts with the fourth serial position, as reference category: that is, we compared late pupil response associated with other serial positions to the late pupil response observed for the fourth serial position: as can be seen on Fig 4C, the late pupil response during the first three serial positions is significantly higher than during the fourth serial position (all *F*s > 6.14, all *p*s < .018, all η_p_^2^s > .15, see green markers on Fig 4C), whereas there was no significant difference between the late pupil response of the fourth and later serial positions ((all *F*s < 1.01, all *p*s > .32, all η_p_^2^s < .03, see grey markers on Fig 4C).

To test whether this effect is related to general factors (e.g. fatigue) or is specific to encoding, we repeated the same analysis also for the recall phase. Because such general effects are supposedly not related to correct retrievals, both correct and incorrect retrieval trials were involved in this particular analysis. We conducted an ANOVA with serial position during the recall phase, as independent variable, and late pupil response accompanying retrieval, as dependent variable. We found no significant effect, *F* (4.41,149.98) = 0.973, *p* = .43, η_p_^2^ = .03. This suggests that the link between serial position and pupil response is specifically related to encoding of items.

Thus, we found that late serial positions (4th to 8th) are associated with an attenuated pupil response. To investigate whether this can serve as a potential confound of our subsequent memory analysis, we compared recall percentage of items encoded in serial positions 1–3 and items encoded in serial positions 4–8. We found that items from the first three serial positions are significantly better remembered than items from later serial positions (*M* = 79.42%, *SE* = 1.3; vs. *M* = 45.78%, *SE* = 4.57; *t*(34) = 5.908, *p* < .001, *d* = 0.99).

**Fig 5:**
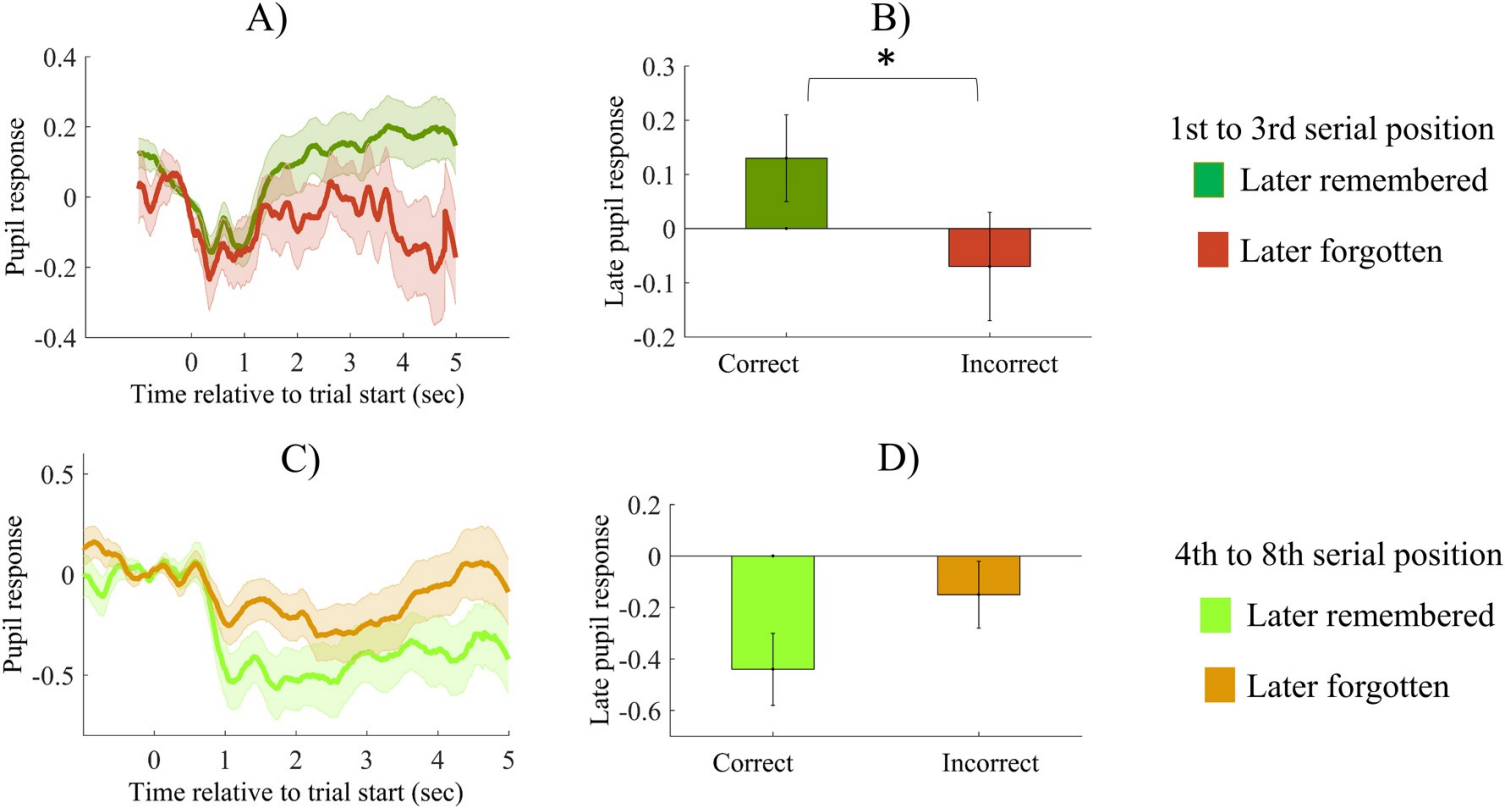
Subsequent memory effects, as a function of serial position. (A)&(C) Pupil size changes related to later recalled and later forgotten items, respectively, depicted separately for the serial positons 1–3 (A) and for serial positions 4–8 (C). Values are standardized and baseline corrected: the mean of the 500 msec preceding trial onset is subtracted from each data point. Time is shown relative to the start of the trial. (B)&(D) Late pupil responses for later recalled and later forgotten items, respectively, depicted separately for the serial positons 1–3 (B) and for serial positions 4–8 (D). Error bars and shaded error regions represent standard error of the mean. * p < .05.

Thus, serial position is related both to increased late pupil response and to recall success. Because of this, it might function as a potential confound. To eliminate this issue, we examined subsequent memory for items from different serial positions. As we found that a decrease in late pupil response is evident after the third serial position, we investigated subsequent memory separately for items encoded on the first three serial positions, and for items encoded on later serial positions. As can be seen on Fig 5, we found that during encoding of the first three items, later successfully retrieved items evoked significantly higher late pupil response, than unsuccessfully recalled items, *t*(34) = 2.25, *p* = 0.03, *d* = 0.38 (Fig 5A and 5B), whereas there was no such effect present for items encoded at late serial positions, *t*(34) = 1.57, *p* = 0.13, *d* = 0.26 (Fig 5C and 5D).

To sum up, we found no significant subsequent memory effect for all items, but our results showed that serial position significantly affects pupil size during encoding: there was a significant decrease in the magnitude of the late pupil response after the encoding of the third item. Because of this, we examined subsequent memory for items encoded in the first three positions versus in later positions, and found that for the first three encoded items, later successful recall is associated with increased late pupil response during encoding.

## Discussion

Here we investigated pupil size changes during encoding and retrieval of paired associates. A pattern of subsequent pupil dilation and constriction was observed during encoding, whereas we found a clear dilation of the pupil during retrieval. The size of the learning set clearly modulated pupil dilation during retrieval. Furthermore, we also found that serial position influences pupil size changes during encoding: pupil dilation during encoding is significantly higher for the first three serial positions, and decreases for later serial positions. Finally, we also found that serial position affects subsequent memory: later remembered items evoke higher pupil dilation for the first three serial positions, whereas this is not the case for later serial positions. In the following, we will go through the results in detail, and also deal with their implication for the pupillometric investigation of episodic memory.

### Pupil size change during encoding and recall: The role of perceptual factors

Interestingly, there was a clear difference between pupil size changes observed during encoding and retrieval. During retrieval, the pupil instantly begun to dilate, as a sign of increased processing load accompanying the retrieval of the target memory. This response was clearly resembling the often showed dilatatory response associated with processing load [3]. In contrast, during encoding, there was a sudden constriction of the pupil, and this was then followed by a dilation. This pattern resembles the pupillary light reflex found in other studies (e.g. [22,24]).

Importantly, there were only minimal luminance changes on the screen between the mask and the stimulus screen, therefore this factor is unlikely to be responsible for the observed constriction. The sudden constriction of the pupil, however, can also be caused by changes of other visual features (e.g. spatial frequency or motion, for a review, see [41]). Furthermore, transient pupil changes accompany the orientation response [39,40], which is the coordinated response of the body triggered by the presentation of novel stimuli or changes in stimulus features [42,43]. Finally, in our task, participants might have lost visual focus during the mask stimulus, and then re-fixated the screen when the word-pairs appeared. This might be accompanied by the accommodation of the eye which also causes pupil constriction [44].

To sum up, the early pupil response observed in our task might be determined by a variety of perceptual factors, which are related to the changes on the screen between the mask and the stimulus screen. The size of the pupil is determined by the relative activity in neural pathways innervating the muscles responsible for the dilation and the constriction of the pupil, respectively (see e.g. [39,40,45]). The balance between these antagonistic systems is influenced by the activity of several subcortical structures, which are sensitive to different perceptual factors (e.g. superior colliculus–pupil orienting response [40,46]; Erdinger-Westphal nucleus–pupillary light reflex [39]). The interactions between these perceptual factors and task characteristics might explain the differences between early pupil size changes associated with encoding and recall, respectively. It might be the case that during retrieval, the initiation of the retrieval process and the verbal, overt response elicits high LC/NA activity, and the resulting pupil dilation overshadows pupil constriction elicited by changes in visual features.

### Memory-strength and retrieval induced pupil dilation

Behavioral results clearly demonstrate that our set size manipulation was successful. Increase in set size was associated with a decrease in accuracy and an increase in retrieval reaction time, suggesting that the size of the learning set influences memory strength of cue-target relationships. In accordance with the results of Van Rijn et al. [32], we found that pupil dilation during retrieval was inversely related to memory strength: larger set size, assumingly associated with weaker cue-target memory strength, was related to larger late pupil response. This result suggests that pupil dilation correlates with processing load of retrieval, which is higher for weaker memories.

Thus, the magnitude of pupil dilation seems to be differently related to memory strength for recognition and cued recall tasks. In a recognition task, manipulations decreasing memory strength (e.g. shallow encoding, see [21]) decrease pupil dilation during recognition. In contrast, for cued recall, our current results and also the results of Van Rijn et al [32] suggest that manipulations decreasing memory strength are associated with larger pupil dilation. These contrary evidence suggest that pupil dilation is not an index of memory strength per se, but it informs us indirectly about memory strength by signaling information processing during a specific memory test. Because memory strength can influence information processing differently for different tests of memory (i.e. cued recall or recognition), the link between memory and pupil size will be determined by the chosen memory task.

One potential factor explaining this difference is the reliance of different memory tasks on automatic, cue-dependent vs. strategic, generative forms of retrieval [47,48]: The former refers to the process whereby a proximal cue automatically and mandatorily triggers the recall of a target memory (this process is also referred to as ecphory, [49,50]. In contrast to automatic retrieval, strategic retrieval is an effortful process involving problem-solving routines which aim to establish effective proximal cues capable of triggering ecphory.

During recognition task, the most effective cue is presented for the participants (the target item itself), thus cue-dependent, automatic retrieval processes might determine performance. In contrast, for a paired associate task, strategic retrieval processes might play a larger role. For example, during encoding, participants might construct a mental image consisting both items, and during retrieval, this image is reconstructed. If the memory is weaker, then such strategic retrieval processes might require more processing resources, and this is evidenced in larger pupil dilation ([3,4,7]). Because of this, recall of weaker memories in a paired associate task will be associated with larger pupil dilation. In contrast, for recognition tasks relying more on automatic retrieval processes, the link between pupil dilation and memory strength might not be determined by processing requirements, but by other factors. For example, it is thought that the link between cue and target is represented in the hippocampus, and this structure activates modality specific parts of the cortex where specific aspects of the target memory (e.g. perceptual, spatial, conceptual) are represented (see e.g. [51]). One might speculate that stronger memories are associated with larger hippocampus triggered cortical activity, and this is accompanied with larger autonomous activity/arousal and larger pupil dilation.

Finally, it is interesting to note that our results show similarity with the seminal findings of Kahneman and Beatty [2], who found that pupil responses during short-term active maintenance of information increases with increasing set size. Our results suggest that such a set-size dependent pupil response can be observed also for the episodic memory system.

### Effects of serial position and later recall success on pupil size changes

As detailed earlier, the sudden constriction at the beginning of encoding (i.e. the early response) is likely induced by perceptual factors. Nevertheless, the pattern of results indicates that the pupil size changes following this sudden constriction (i.e. the late pupil response) are also affected by higher-level cognitive factors. First, we found that serial position modulated pupil size changes during encoding: Late pupil response was higher during the encoding of the first three word-pairs, as compared to the encoding at later serial positions. Importantly, no such effect was demonstrated for the test phase, which suggests that this pattern is specifically related to encoding processes, and cannot be attributed to general factors associated with serial processing of subsequent items (e.g. fatigue).

Furthermore, we found no significant subsequent memory effect in our sample, but we demonstrated that this is due to a modulation of serial position: in line with our predictions, for the first three serial positions, the encoding of later recalled items was accompanied by larger late pupil response than the encoding of later forgotten items. However, in contrast to our prediction, for items encoded on late serial positions (4th to 8th), we found no significant difference between later recalled and forgotten items, respectively (and the encoding of forgotten items triggered a nominally higher pupil response).

This pattern suggests that qualitatively different processes play a role during encoding of items from early and late serial positions, respectively. During encoding of early word-pairs, participants might try to rehearse the current and previous word-pairs, and so the working memory system might become gradually overloaded. In previous research, task-evoked pupil dilations were shown to decrease once the amount of information exceeded the capacity limit of the working-memory system [52,53]. Thus, the decrease in pupil dilation after the encoding of the third word-pair might signal the overloading of the working memory system, and so effortful rehearsal of items is only efficient during the encoding of the first three items. This explanation is also in line with our subsequent memory findings: for the first three serial positions, successful encoding and later recall is predicted by high attentional effort and so large pupil response, whereas unsuccessful encoding and later forgetting is predicted by attentional slips and inattentiveness, and so smaller pupil dilation. For the late serial positions, effortful rehearsal of previous items is not possible anymore, and participants might switch to a different encoding strategy, and so the magnitude of pupil dilation is not predictive of recall success anymore.

A further possibility might be the influence of another neurotransmitter, acetylcholine (ACh). The pupil constrictor muscle is controlled by cholinergic preganglionic neurons, and ACh is involved in the regulation of the most frequent constrictive pupil size change, the pupillary light reflex [4,39, 54]. Furthermore, high levels of hippocampal ACh are thought to reduce the interference from previously encoded information, and this promotes encoding, particularly under conditions with high interference [55,56]. Based on the link between ACh, memory encoding and novelty, Kafkas and Montaldi [57] suggested that pupillary constriction during novelty detection might be associated with cholinergic mechanisms, and this possibility was also raised by others [24]. Consequently, in our task, interference from previously encoded items might result in cholinergic efflux, and this might be responsible for the diminished dilation of the pupil in late encoding trials. It must be noted, however, that there is also evidence contrasting such explanation. For example, results showing cholinergic modulation of the pupillary reflex can also originate from effects involving the peripheral nervous system [58]. Furthermore, in a recent paper [59], it was shown that cortical cholinergic activity in rats is associated with pupil size increase (and not decrease). Thus, before a potential link between ACh, memory and pupil size changes can be revealed, the influence of central and peripheral cholinergic effects on pupil size has to be further investigated.

To sum up, we found that both serial position and later recall success was associated with pupil size changes during encoding of word-pairs. The fact that serial position modulated the effects of recall success draws attention to the fact, that encoding can be quite variable: several mental processes might be involved in the encoding of items, which trigger different cognitive and neurobiological responses, and these responses differently affect pupil size. This might explain the inconsistent subsequent memory effects in recognition memory literature in general [20,24,27].

### Limitations

First, it is important to note, that in terms of retrieval-related pupil size changes only the difference between the large and the medium set size was significant, whereas there was no significant difference between the pupil responses evoked by the small and the medium set sizes. This might be caused by the nature of our set size manipulation. Set size increased nonlinearly, therefore the difference between the medium and the large set size was larger than the difference between the small and the medium set sizes. It is possible that pupil dilation is not sensitive enough to show the processing load differences between the small and the medium set sizes. Alternatively, retrieval from the large set size condition involves qualitatively different processes, and this is manifested in the larger pupil dilation. For example, when treating the word-pairs as chunks, two or four word-pairs can be maintained also in short-term memory [60], consequently retrieval from these learning sets might rely on short-term memory processes, whereas retrieval from the large set size might require long-term retrieval. This alternative explanation is unlikely, however, as the demanding computation task during the delay period makes it unlikely that the word-pairs could be maintained in working memory. Further studies with a more thorough manipulation of set size might shed light on this issue.

Second, the 20 second delay between encoding and recall was relatively short compared to other investigations of episodic memory. It was intended to prohibit the active maintenance of the information, so the recall task relies only on the reactivation of the episodic memory traces, and not retrieval from short-term memory buffers. Nevertheless, consolidation processes on a larger time scale (hours to days) play also a crucial role in episodic memory processes [61,62], thus a more complete picture of pupil size changes during cued recall would require a paradigm with longer retention delays.

Third, during word selection, we did not control for two factors known to influence recall performance: word imageability and emotionality. Whereas this is an obvious limitation of our work, most of the analyses are not affected: Word-pairs were randomly assigned to the different set-size conditions, thus differences between the word-pairs cannot cause systematic differences between the conditions. There is one particular analysis, however, were these uncontrolled factors might play a third-variable role. For the subsequent memory analysis, word-pairs were assigned to the conditions based on recall success: here the link between pupil dilation and recall success can be caused by word imageability and/or emotionality (i.e. imageable or emotionally valenced words evoke higher pupil dilation during encoding and are better remembered later). It is important to note, however, that we excluded words with obvious emotional content, and most of the words were nouns representing imageable objects (see word list in S1 Appendix). Thus we argue that word imageability and emotionality are not potent mediator factors in our design: nevertheless, further research is required to determine the possible role of these factors in the causal link between increased pupil dilation and later recall success.

## Conclusion

Our study was the first to investigate pupil size changes during both encoding and retrieval of paired associates. We showed that the encoding and retrieval of paired associates are associated with a different pattern of pupil size changes. Some of these changes are determined by visual/perceptual factors, but others are associated with high level cognitive factors, such as encoding position or size of the learning set. This suggests that pupil size changes are sensitive to the complex dynamics of episodic encoding and retrieval, and further research might gain valuable insight regarding the neurobiological determinants of these processes.

## Supporting information

S1 Appendix List of Hungarian word-pairs used in the experiment. English translations are in parenthesis.
(DOCX)

S1 Text RT data analysis without participants with high RT data loss. (DOCX)

S1 Dataset (XLSX)

S2 Dataset (XLSX)
