# Supplementary material for: How the size of the to-be-learned material influences the encoding and later retrieval of associative memories: A pupillometric assessment
Source: PLoS One. 2019 Dec 31;14(12):e0226684. doi: 10.1371/journal.pone.0226684 (PMC6938364; doi:10.1371/journal.pone.0226684)
Supplement: S1 Appendix — English translations are in parenthesis. (DOCX) [file pone.0226684.s001.docx]

**Appendix 1**. List of Hungarian word-pairs used in the experiment. English translations are in parenthesis.

| **CUE WORD** |  | **TARGET WORD** |
| --- | --- | --- |
|  |  |  |
| apród (henchman) | - | rosta (sieve) |
| bukta (tart) | - | garat (pharynx) |
| zúgás (buzzing) | - | kőris (ash) |
| gerle (dove) | - | menza (canteen) |
| horda (horde) | - | orkán (gale) |
| rámpa (ramp) | - | tájék (region) |
| magma (magma) | - | jászol (manger) |
| tubus (tube) | - | korpa (bran) |
| cékla (beetroot) | - | lugas (arbor) |
| sóska (sorrel) | - | pedál (pedal) |
| fivér (brother) | - | tövis (thorn) |
| horog (hook) | - | támla (backrest) |
| sarló (sickle) | - | mamut (mammoth) |
| korzó (promenade) | - | nektár (nectar) |
| teflon (teflon) | - | hárfa (harp) |
| kuckó (nook) | - | polip (octopus) |
| zombi (zombie) | - | palló (plank) |
| jurta (yurt) | - | cirkó (circulating) |
| butik (boutique) | - | köret (garnish) |
| hurka (sausage) | - | bádog (tin) |
| babér (laurel) | - | kánon (canon) |
| juhar (maple) | - | perec (pretzel) |
| kefír (kefir) | - | fonál (thread) |
| huzal (wire) | - | tónus (tone) |
| bácsi (uncle) | - | morzsa (crumb) |
| motel (motel) | - | emlős (mammal) |
| felni (rim) | - | számla (invoice) |
| bogár (bug) | - | fohász (prayer) |
| hőtan (thermodynamics) | - | bölény (bison) |
| randi (date) | - | brácsa (viola) |
| kupak (cap) | - | lepény (pie) |
| dohány (tobacco) | - | zsivaly (noise) |
| intés (beck) | - | kócsag (heron) |
| karám (pinfold) | - | osztag (squad) |
| kenet (unction) | - | tapasz (patch) |
| szeder (mulberry) | - | arzén (arsenic) |
| bicska (pocket knife) | - | fuvar (ride) |
| csukló (wrist) | - | sumér (sumer) |
| nyaláb (sheaf) | - | pacal (tripe) |
| gyapot (cotton) | - | kajak (kayak) |
| hiszti (tantrum) | - | arány (proportion) |
| kedély (temper) | - | nemes (noble) |
| kenőcs (paste) | - | pocok (gopher) |
| szabás (cut) | - | peták (dime) |
| szifon (siphon) | - | fogoly (prisoner) |
| csupor (jar) | - | főcím (headline) |
| kezes (guarantor) | - | gallér (collar) |
