# Supplementary material for: How the size of the to-be-learned material influences the encoding and later retrieval of associative memories: A pupillometric assessment
Source: PLoS One. 2019 Dec 31;14(12):e0226684. doi: 10.1371/journal.pone.0226684 (PMC6938364; doi:10.1371/journal.pone.0226684)
Supplement: S1 Text — (DOCX) [file pone.0226684.s002.docx]

**RT data analysis without participants with high RT data loss**

Due to problems in recording reaction times, for five participants no reliable reaction time data could be recorder for at least 15 trials. To prove that this data loss does not contribute to the observed effect of set size on reaction time of recall, we repeated the ANOVA presented in the main text after exclusion of this five participants. The pattern of results does not change. The main effect of set size is significant, F (1.2,35.9) = 22.11, p < .001, ηp2 = .43, as dependent variables. Planned contrast analyses showed that the three set size conditions differed from each other significantly (small vs. medium: F [1,29] = 96.9, p < .001, ηp2 = .77; medium vs. large: F [1,34] = 21.57, p < .001, ηp2 = .43).
